# The *Plasmodium berghei* merozoite protein *Pb*GAC is critically involved in erythrocyte binding during invasion

**DOI:** 10.1186/s13071-025-07067-5

**Published:** 2025-10-28

**Authors:** Junying Gao, Ning Jiang, Yiwei Zhang, Ran Chen, Ying Feng, Xiaoyu Sang, Qijun Chen

**Affiliations:** 1 Key Laboratory of Livestock Infectious Diseases, Ministry of Education, Key Laboratory of Zoonosis, College of Animal Science and Veterinary Medicine, Shenyang Agricultural University, Shenyang, Liaoning China; 2 Research Unit for Pathogenic Mechanisms of Zoonotic Parasites, Chinese Academy of Medical Sciences, Shenyang, Liaoning China

**Keywords:** *Plasmodium*, Heparin-binding proteins, Invasion, Pathogenicity

## Abstract

**Background:**

The invasion of *Plasmodium* merozoites into host erythrocytes is initiated through specific ligand–receptor interactions. This interaction results in subsequent invasion events, facilitated by the formation of a moving junction via AMA-1 and associated molecular complexes. Previous studies have implicated erythrocyte surface glycosaminoglycans, particularly heparan sulfate proteoglycans, as critical receptor components in this invasion process.

**Methods:**

The binding affinity of the *Pb*GAC protein to heparin and erythrocytes was assessed through western blotting, immunofluorescence, flow cytometry techniques, and heparinase II treatment. Mice were immunized with the recombinant *Pb*GAC-His to generate specific polyclonal antibodies for subcellular localization, passive immunization, and immunoprecipitation. Global mass spectrometric analyses were conducted to identify its interacting proteins.

**Results:**

We elucidated the molecular function of *Pb*GAC (encoded by *Pb*ANKA_1137800), a previously uncharacterized *Plasmodium berghei* ANKA protein, in association with merozoite attachment and invasion via the heparan sulfate-dependent pathway. The *Pb*GAC protein, predominantly located at the extreme apical region of the *P. berghei* merozoite, binds to heparin and the erythrocyte surface during merozoite invasion. Global mass spectrometric analysis reveals that *Pb*GAC interacts with several secreted proteins that are critically involved in erythrocyte invasion. In addition, mice either immunized with the *Pb*GAC protein or passively immunized with sera derived from vaccinated mice demonstrated enhanced immunity against lethal challenges.

**Conclusions:**

Our findings pinpointed that *Pb*GAC is predominantly expressed at the extreme apical region of the *P. berghei* merozoite and engaged in binding to the heparin-like receptors on the erythrocyte surface during merozoite invasion.

**Graphical Abstract:**

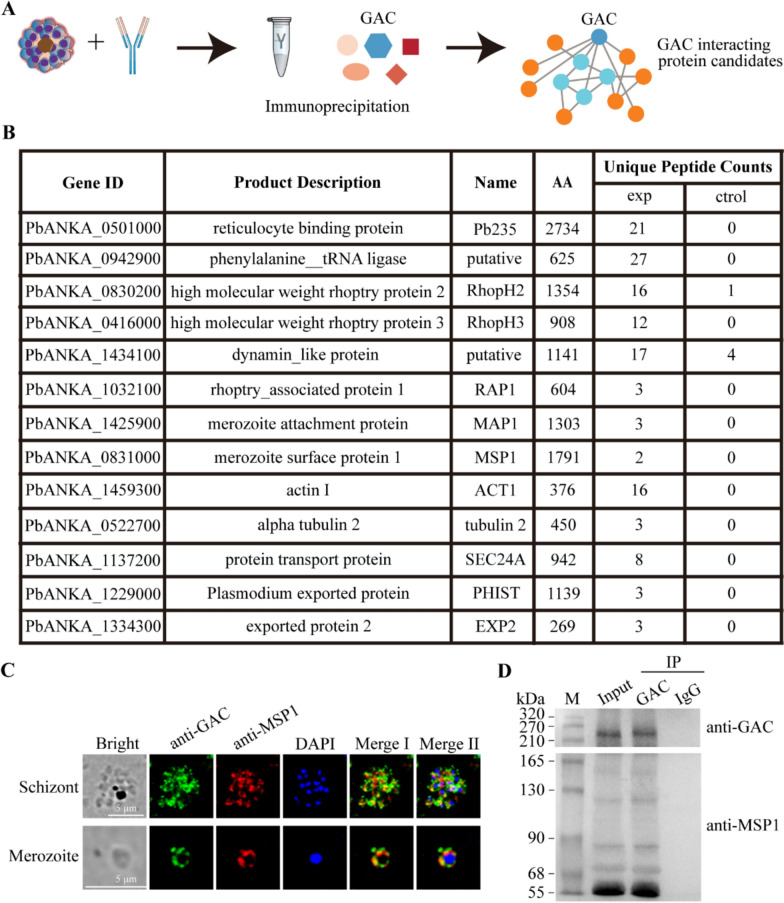

**Supplementary Information:**

The online version contains supplementary material available at 10.1186/s13071-025-07067-5.

## Background

Members of the phylum Apicomplexa constitute a group of obligatory intracellular protozoan parasites responsible for major human parasitic diseases, including malaria and toxoplasmosis. *Plasmodium* species exhibit a complex biphasic life cycle involving asexual replication in vertebrate hosts and sexual development in mosquito vectors [1]. The pathogenesis of severe malaria is directly correlated with the exponential expansion of asexual blood-stage parasites, where cyclical fever episodes coincide with the synchronized egress of merozoites from lysed erythrocytes [2]. During intraerythrocytic development, invasive merozoites progress through morphologically distinct stages—ring, trophozoite, and schizont—with mature schizonts generating 16–32 daughter merozoites capable of initiating new infection cycles [3]. To bypass the erythrocyte barrier, *Plasmodium* merozoites utilize a distinctive mechanism for erythrocyte invasion. This process begins with a “long-distance” recognition of surface receptors, which is succeeded by a reorientation phase that sustains these low-affinity interactions. Once the apical end of the merozoite is in proximity to the erythrocyte, a tight junction is established through high-affinity ligand–receptor interactions. Subsequently, this tight junction translocates from the apical to the posterior pole, driven by the actin–myosin motor of the parasite [4]. This erythrocytic invasion process, governed by specialized invasion-associated proteins, represents an essential mechanism for parasite propagation.

The *Plasmodium* rapid replicative cycle requires coordinated gliding motility to facilitate host-cell entry, egress, and traversal through biological barriers [5–7]. Merozoite invasion of erythrocytes occurs through four mechanistically distinct phases: (1) low-affinity attachment to the erythrocyte membrane, (2) apical reorientation, (3) formation of a tight moving junction, and (4) establishment of the parasitophorous vacuole [8]. At each stage, the process involves the kinetics of merozoite invasion and elucidates the ligand–receptor interactions involved. A series of well-characterized merozoite ligands are sequentially released from merozoite organelles, specifically rhoptries and micronemes, facilitating a tight attachment and reorientation of the merozoite toward its apical end on the erythrocyte surface in preparation for invasion. At the molecular level, these preinvasion events initiate when merozoite surface proteins (MSPs) mediate the initial, low-affinity attachment, which subsequently triggers the expression of erythrocyte binding-like (EBL) and reticulocyte binding-like (RBL) family members as well as apical membrane antigen (AMA-1) on the merozoite surface [9–12]. Initial merozoite–erythrocyte interactions, primarily mediated by glycosylphosphatidylinositol (GPI)-anchored surface proteins, are critical for invasion competency [13]. Key molecular mediators include merozoite surface protein 1 (MSP1), thrombospondin anonymous repeat protein (TRAP), erythrocyte binding-like protein 175 (EBA-175), and reticulocyte-binding protein 4 (PfRh4), which collectively facilitate erythrocyte adhesion [14–18]. The binding of Rh5 to basigin (BSG), a blood group antigen of the Ok blood group system, is an essential binding event, representing the irreversible commitment step for merozoite entry into the erythrocyte and acting as the trigger for all subsequent invasion processes [19]. The Rh5-binding event promotes the formation of a fusion pore between the merozoite rhoptries and the erythrocyte, initiating calcium influx into the erythrocyte and the release of rhoptry contents [20]. Subsequent invasion steps involve proteins derived from apical organelles, such as apical membrane antigen 1 (AMA-1), a type 1 transmembrane protein, which is secreted onto the merozoite surface during egress from the previous host cell. AMA-1 interacts with RON2 to establish the moving junction complex, a critical structure mediating parasite internalization [21–23].

Heparan sulfate (HS), a ubiquitous glycosaminoglycan on vertebrate cell surfaces, serves as a key binding partner for apicomplexan surface antigens and secretory proteins [24–26]. Although HS-binding proteins remain incompletely characterized, current evidence identifies *Toxoplasma gondii* SAG1, ROP2/4, and GRA2 as invasion-related HS interactors [27]. In *Plasmodium*, merozoite surface protein *Pf*MSP-1 and merozoite attachment protein *Pb*MAP1 demonstrate HS-binding capacity [18, 24, 28]. Notably, apical organelle-localized proteins also engage specific erythrocyte receptors during invasion [29, 30]. The *Toxoplasma* glideosome-associated connector (GAC) exemplifies this paradigm, coordinating actin dynamics, microneme protein interactions (MIC2), and phosphatidic acid signaling to regulate gliding motility and invasion. *Pf*GAC is also associated with parasite actin to facilitate invasion [31, 32]. However, the functional interplay between plasmodial GAC homologs and HS receptors remains to be elucidated.

This study elucidates the invasion-related functions of *Plasmodium berghei* glideosome-associated connector (*Pb*GAC; *Pb*ANKA_1137800). We demonstrate that *Pb*GAC exhibits specific HS-binding activity and that *Pb*GAC-specific antibodies confer invasion inhibition in murine models. Immunoprecipitation analyses further reveal its association with merozoite invasion machinery. These findings establish *Pb*GAC as a critical mediator of erythrocyte invasion in *P. berghei*.

## Methods

### Animals

Female BALB/c mice (2 weeks old) and Sprague–Dawley rats (2 weeks old) were purchased from Liaoning Changsheng Biological Technology Company (Benxi, China). Animal experiments were carried out under the institutional guidelines of the Shenyang Agricultural University, China (ethical approval no. SYXK < Liao > 2021-0010).

### Sequence and phylogenetic analysis of *Pb*GAC

The gene sequence of *Pb*GAC (*Pb*ANKA_1137800) and orthologs from other *Plasmodium* species were retrieved from the malaria database (https://plasmodb.org). Multiple sequence alignment of *Pb*GAC was performed using DNAMAN software [33]. Phylogenetic analysis of *Pb*GAC orthologs was conducted using MEGA 11 [34].

### Protein expression and purification

The *Pb*GAC (amino acids 99–297) amplified products were cloned into the *Bam*H I/*Xho* I sites of the pET-28a and pGEX-4T-1 expression vectors to produce recombinant *Pb*GAC-His and GST-*Pb*GAC proteins. Primers used for cloning the coding sequence are provided in Additional File 1: Supplementary Table S1. The plasmids were transformed into BL21 (DE3) or BL21 *Escherichia coli* strains for protein expression. The *Pb*GAC-His protein was purified via Ni^2+^ affinity chromatography, while GST-*Pb*GAC was purified through glutathione affinity chromatography. The two recombinant proteins were analyzed by sodium dodecyl sulphate–polyacrylamide gel electrophoresis (SDS–PAGE) and western blotting with tag-specific antibodies as described [18].

### Generation of *Pb*GAC-specific antibodies

The purified *Pb*GAC-His protein was used to immunize rats (*n* = 3) and mice (*n* = 10). For the initial immunization, *Pb*GAC-His protein was emulsified with Freund’s complete adjuvant, and doses of 50 μg per mouse and 100 μg per rat of *Pb*GAC-His protein were administered subcutaneously. This was followed by three additional immunizations, where *Pb*GAC-His protein was emulsified with incomplete Freund’s adjuvant at the same dosages, with a 14-day interval between each administration. Following the final immunization, sera were collected on day 7. Antibody titers were assessed using enzyme-linked immunosorbent assay (ELISA) with recombinant GST-*Pb*GAC as the coating antigen [18]. Immunoglobulin G (IgG) was purified from rat sera utilizing Protein G Sepharose 4 Fast Flow Resin (Cytiva, USA) according to the manufacturer’s protocol. Briefly, 200 μL of Protein G magnetic beads were transferred to a 1.5-mL Eppendorf (EP) tube, followed by the addition of 1 mL of 20 mM sodium phosphate, ensuring a thorough resuspension of the beads. The mixture was centrifuged at 500*g* for 5 min at 4 °C, and the wash step was repeated twice. The serum was mixed with 20 mM sodium phosphate buffer in a 1:2 ratio, then incubated with the Protein G magnetic beads at 4 °C for 2 h. Subsequently, 1 mL of 20 mM sodium phosphate buffer was added, and the beads were resuspended, centrifuged, and the supernatant discarded; this wash step was repeated four times. Finally, 1 mL of 0.1 mM citric acid was added to the Protein G magnetic beads and incubated at 4 °C for 10 min, followed by centrifugation, and the supernatant was collected into a new EP tube. Immediately, 200 μL Tris-HCL (pH 9.0) was added to neutralize the eluate.

### Localization of *Pb*GAC in *P. berghei* via an immunofluorescence assay

*Plasmodium berghei*-infected erythrocytes were harvested directly from the blood of infected mice. Smears were fixed with pure methanol for 10 min at room temperature. The cells were permeabilized with 0.1% Triton X-100 for 10 min and blocked with 3% bovine serum albumin (BSA) at 37 ℃ for 1 h. Cells were incubated overnight at 4 ℃ with anti-*Pb*GAC-specific antibodies (1:300). Following incubation, the cells were washed five times with phosphate-buffered saline (PBS) and then incubated with a secondary anti-rat Alexa Fluor 488 antibody (1:500, Thermo Fisher Scientific, USA) for 30 min at 37 °C, followed by five additional washes with PBS. The cells were mounted using ProLong Diamond Antifade Mountant with DAPI (Thermo Fisher Scientific, USA) and visualized under a fluorescence microscope (Leica, Germany).

### Heparin-binding and competition assays

Purified GST-*Pb*GAC and GST proteins (200 μg) were respectively mixed with heparin–sepharose beads (50 μL) (Cytiva, USA) and incubated for 2 h at 4 °C in an inverted mixer. The samples underwent seven washes with PBS and were centrifuged at 500*g* for 5 min at 4 ℃. The samples were then combined with 50 μL of 1× SDS–PAGE loading buffer and heated at 100 °C for 5 min. After brief centrifugation, the supernatant was collected for SDS–PAGE analysis.

To further assess the specificity of GST-*Pb*GAC binding to heparin, GST-*Pb*GAC was incubated with varying concentrations of heparin and chondroitin sulfate (CSA) (10, 1, 0.1, 0.01, 0.001, and 0.0001 mg/mL) for 30 min. Subsequently, 40 μL of heparin–agarose beads were gradually added to the mixture and incubated at 4 °C for 2 h. The samples were washed seven times with PBS and centrifuged at 500*g* for 5 min at 4 °C. The competition effect of heparin and CSA on the binding of GST-*Pb*GAC to the heparin–agarose beads was analyzed using western blotting.

### Binding of recombinant *Pb*GAC to mouse erythrocytes

GST-*Pb*GAC and GST (200 μg, 200 μL) were respectively incubated with mouse erythrocytes (7 μL packed volume) in PBS for 1 h at 37 °C (final volume 1 mL) as previously described [35]. The erythrocytes were washed seven times with PBS and resuspended in 30 μL 1× SDS–PAGE loading buffer, followed by heating at 100 °C for 5 min. The protein was resolved in a 10% acrylamide gel and transferred to the polyvinylidene difluoride (PVDF) membrane as described [18]. The membrane was probed with the anti-GST mAb (1:3,000, TransGen, China), followed by the horseradish peroxidase (HRP)-conjugated goat anti-mouse IgG (H+L) secondary antibody (1:5,000, Beyotime, China). The protein bands were visualized via enhanced chemiluminescence detection.

To further validate the binding of *Pb*GAC to mouse erythrocytes, freshly collected erythrocytes were washed with PBS, and blood smears were prepared and fixed with methanol for 30 s. The fixed erythrocytes were washed in PBS, blocked with 3% bovine serum albumin (BSA) for 1 h at 37 °C, washed again (five times, 10 min each), and incubated with 200 μg GST-*Pb*GAC or GST in 3% BSA for 2 h at 37 °C. The slide samples were washed five times with PBS and incubated with goat anti-GST antibody (1:3,000, TransGen, China), followed by five washes and incubation with Alexa Fluor 488 goat anti-mouse IgG (H + L) (1:500, Thermo Fisher Scientific, USA). High-resolution images were captured using a fluorescence microscope (Leica, Germany), and the mean fluorescence intensity of the erythrocytes was determined using the FACSAria III flow cytometer (BD Biosciences, USA).

Some erythrocytes were treated with heparinase II (0, 1, and 5 U/mL) in Roswell Park Memorial Institute (RPMI)-1640 for 1 h at 37 °C, washed three times in PBS, and incubated with 200 μg GST-*Pb*GAC or GST. Proteins on erythrocytes were detected using western blotting.

### Calcium ionophore-induced *Pb*GAC translocation

*P. berghei*-infected erythrocytes were cultivated in vitro overnight as previously described [36]. Freshly egressed merozoites were subjected to two washes with Dulbecco’s modified Eagle medium (DMEM), then resuspended in DMEM containing 30 μM R59022 (DGK inhibitor) and placed on coverslips within a six-well plate at room temperature for 30 min [31]. After pretreatment, parasites were incubated with 6 μM A23187 (calcium ionophore) for 15 min at 37 ℃ in a 5% CO_2_ atmosphere. Following incubation, samples were fixed for 10 min with methanol. The fixed cells were permeabilized with 0.1% Triton X-100 for 10 min, followed by blocking at 37 ℃ for 1 h with 3% BSA. Subsequently, the cells were incubated overnight at 4 ℃ with anti-*Pb*GAC antibodies diluted in 3% BSA and then treated with a secondary anti-rat Alexa Fluor 488 antibody (1:500, Thermo Fisher Scientific, USA) for 30 min at 37 °C, after which they were washed five times with PBS. The cells were mounted using ProLong Diamond Antifade Mountant with DAPI (Thermo Fisher Scientific, USA) and visualized under a fluorescence microscope (Leica, Germany).

### *Pb*GAC secretion assay

To prepare parasite culture supernatant, purified schizonts were incubated in the absence of red blood cells (RBCs) at 37 °C for 5 min with 2% ethanol [37]. Subsequently, the suspension was placed in an ice bath for 10 min to promote further secretion. Cells were centrifuged at 12,000 rpm for 10 min to collect the supernatants. The supernatants were directly subjected to 6% SDS–PAGE. Following electrophoresis, gels were transferred onto PVDF membranes (Bio-Rad). Specific proteins were detected using *Pb*GAC-specific antibodies (1:500), followed by HRP-conjugated secondary antibodies (1:10,000) and enhanced chemiluminescence (Beyotime, China).

### Immunoprecipitation and mass spectrometry

Synchronized *P. berghei* were harvested at the schizont stage via centrifugation at 400*g* for 20 min [38]. The parasites were pelleted, washed in cold PBS, and subjected to centrifugation at 14,000*g* for 10 min at 4 ℃ to eliminate hemoglobin. The samples were lysed using NP-40 lysis buffer (Beyotime, China) containing 1% protease inhibitor phenylmethylsulfonyl fluoride (PMSF) for 30 min on ice, followed by centrifugation at 12,000 rpm for 10 min at 4 ℃. The supernatants of the parasite lysates were split into two portions, with one portion incubated with the rat anti-*Pb*GAC antibodies and another incubated with normal rat IgG overnight at 4 ℃ on an inverted mixer. Subsequently, the samples were respectively combined with protein G-conjugated magnetic beads and incubated for 4 h at 4 ℃ on an inverted mixer. Finally, the beads were washed five times with cold PBS and resuspended in 100 μL 1× SDS–PAGE loading buffer, heated at 100 °C for 5 min. The beads were sedimented by brief centrifugation, and the supernatants were separated by SDS–PAGE. After electrophoresis, the gels were stained with Coomassie Brilliant Blue solution. The protein bands were sliced from the gels and subjected to trypsinization and mass spectrometry analysis as previously described [39].

### Immunization with *Pb*GAC-His protein and challenge assay

For the initial immunization, *Pb*GAC-His protein emulsified in complete Freund’s adjuvant was administered subcutaneously at a dosage of 50 μg per mouse (*n* = 10). This was followed by three additional immunizations with 50 μg *Pb*GAC-His protein emulsified in incomplete Freund’s adjuvant, with a 14-day interval between each administration. The ELISA was performed to quantify the antibody titers with GST-*Pb*GAC as the coating antigen as described previously [18]. Once the antibody titer reached 1:32,000, 1 × 10^6^ parasitized erythrocytes per mouse were intraperitoneally injected. Peripheral blood parasitemia was evaluated using Giemsa-stained thin blood smears, and the survival duration of the mice was monitored.

### Passive immunization assays with *Pb*GAC-immune sera

A total of 20 mice were randomly allocated into two groups. On the initial day, each mouse received an injection of the immune serum (0.5 mL per mouse) via the tail vein. On the subsequent day, each mouse was administered 1 × 10^6^ infected erythrocytes through an intraperitoneal injection. On the third day, each mouse was intravenously injected with the same amount of the immune serum. The assessment of peripheral blood parasitemia was conducted using Giemsa-stained thin blood smears, and the survival duration of the mice was monitored.

## Results

### Sequence and phylogenetic analysis of *Pb*GAC

**Fig. 1:**
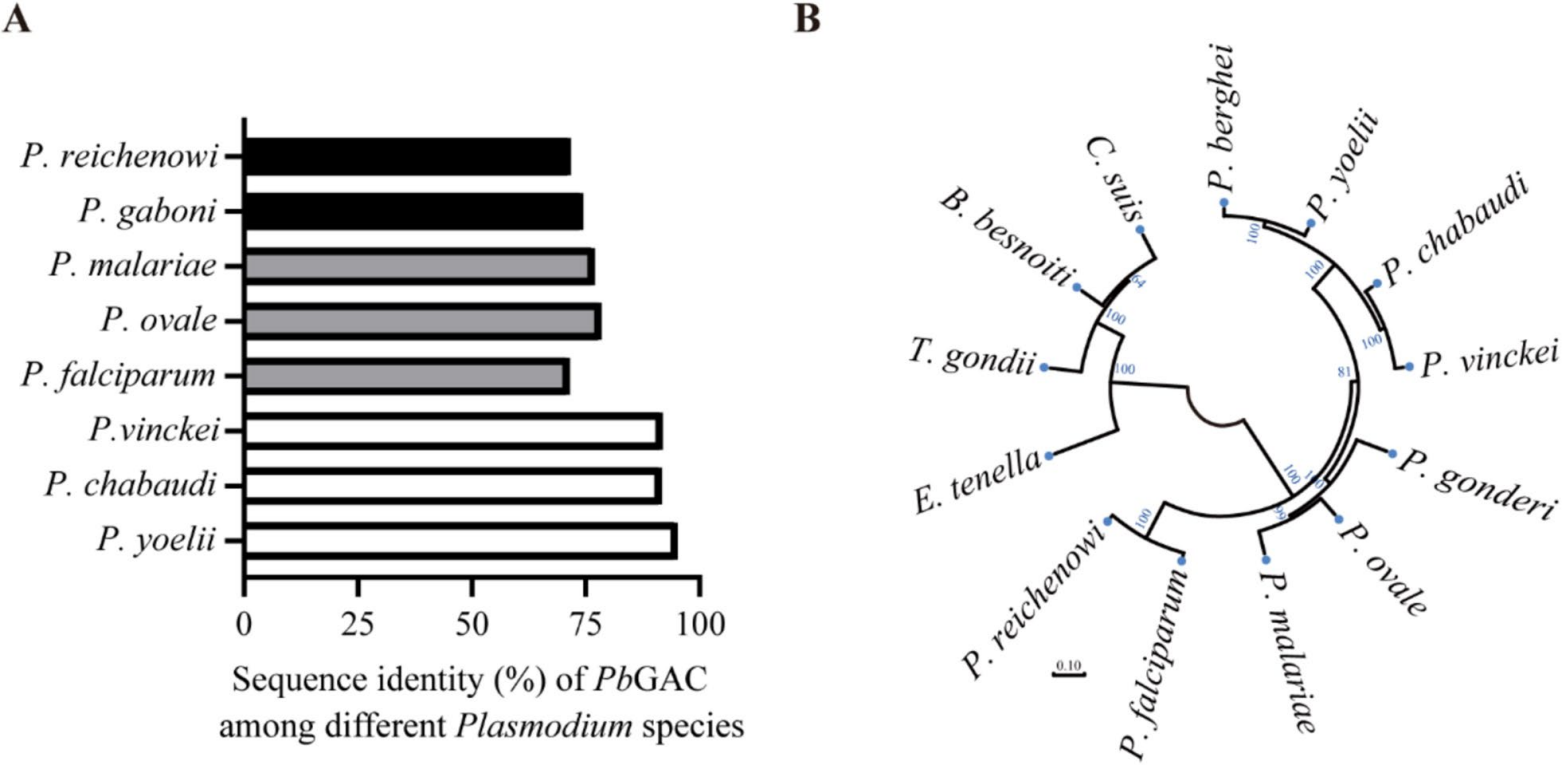
Bioinformatic analyses of *Pb*GAC and homologous proteins from other parasites. **A**, Percentage identity of the GAC sequence from *P. reichenowi*, *P. gaboni*, *P. malariae*, *P. ovale*, *P. falciparum*, *P. vinckei*, *P. chabaudi*, *P. yoelii*, and *P. berghei*. **B**, The unrooted phylogenetic tree was inferred from the GAC alignment. The tree was constructed using the neighbor-joining algorithm, on the basis of a hidden Markov model (HMM) multiple alignment. Bootstrap values are shown in blue. The reliability of the branches was assessed by the bootstrap resampling with 1000 replicates

Here, we found that the amino acid sequence of *Pb*GAC is highly conserved among *Plasmodium* spp., with > 90% identity between *P. yoelii*, *P. chabaudi*, and *P. vinckei* orthologs and > 70% identity across species infecting humans and apes (Fig. 1a). From an evolutionary perspective, *Pb*GAC is closely related to *P. yoelii* and emerged later than *T. gondii* (Fig. 1b).

### *Pb*GAC is expressed during the asexual development of *P. berghei*

**Fig. 2:**
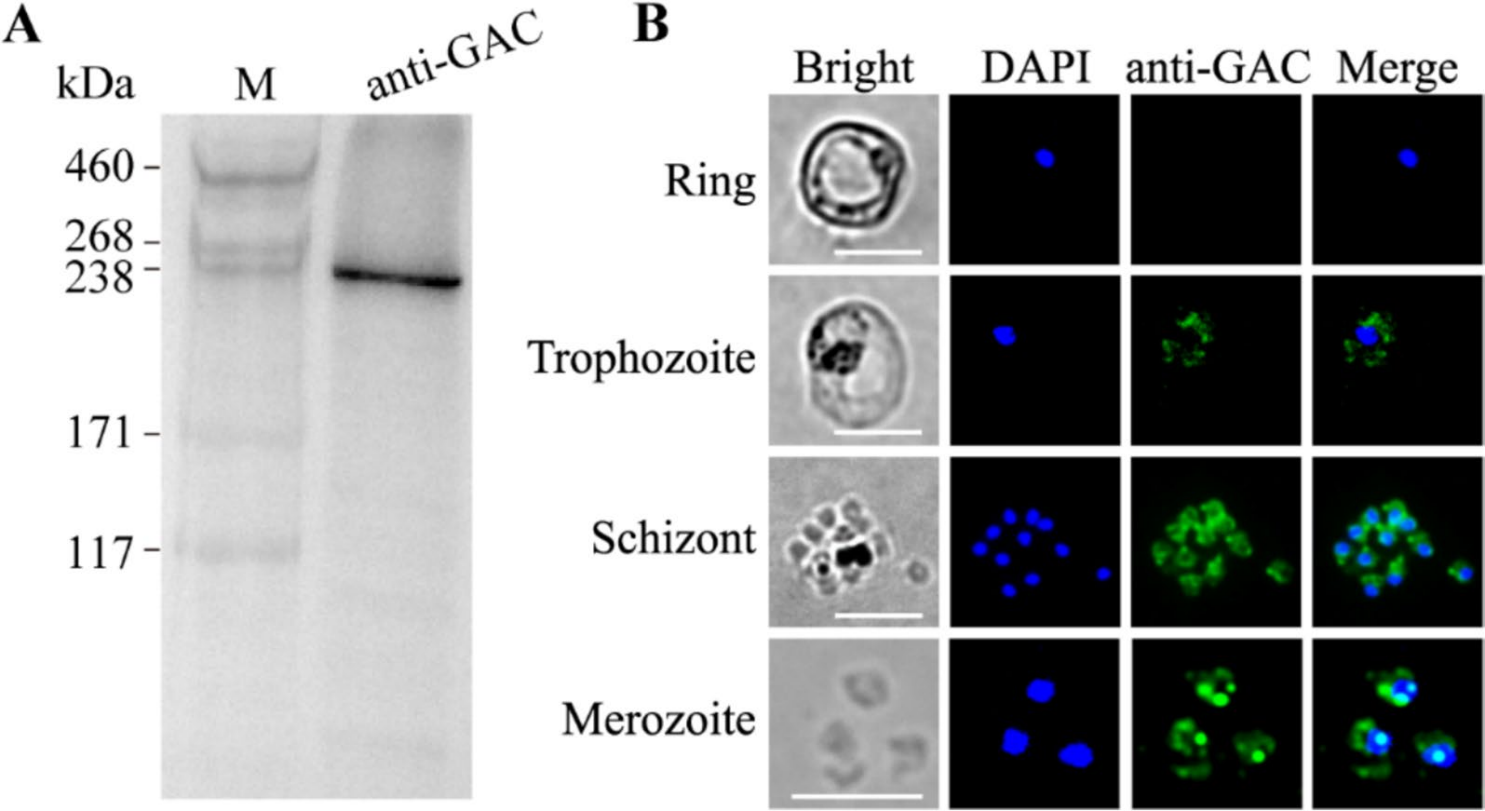
*Pb*GAC is mainly expressed in the apical region and cytosol. **A**, Western blotting analysis of native *Pb*GAC expressed in *P. berghei* merozoites detected using *Pb*GAC-specific antibodies. **B**, Indirect immunofluorescence of *Pb*GAC was performed on free merozoites and parasites at ring, trophozoite, and schizont stages, with *Pb*GAC expression detected using anti-*Pb*GAC IgG (green). Parasite nuclei were stained with DAPI (blue). Scale bar, 5 μm

To elucidate the localization of the *Pb*GAC, we generated specific antibodies against *Pb*GAC by immunization with HIS-tagged *Pb*GAC recombinant protein. The antibody specifically recognized the native *Pb*GAC in western blotting (Fig. 2a). Furthermore, indirect immunofluorescence assays (IFA) were performed to explore the subcellular localization of *Pb*GAC in asexual-stage parasites. Our observations indicated that *Pb*GAC was undetectable in the ring stage and appeared from the trophozoite stages. During the blood-stage merozoite invasion, *Pb*GAC localized to the cytosol, exhibiting a distinct accumulation at the extreme apical region, suggesting a pivotal role for *Pb*GAC in merozoite function (Fig. 2b).

### The distribution of *Pb*GAC in the parasite was modulated by calcium ions

**Fig. 3:**
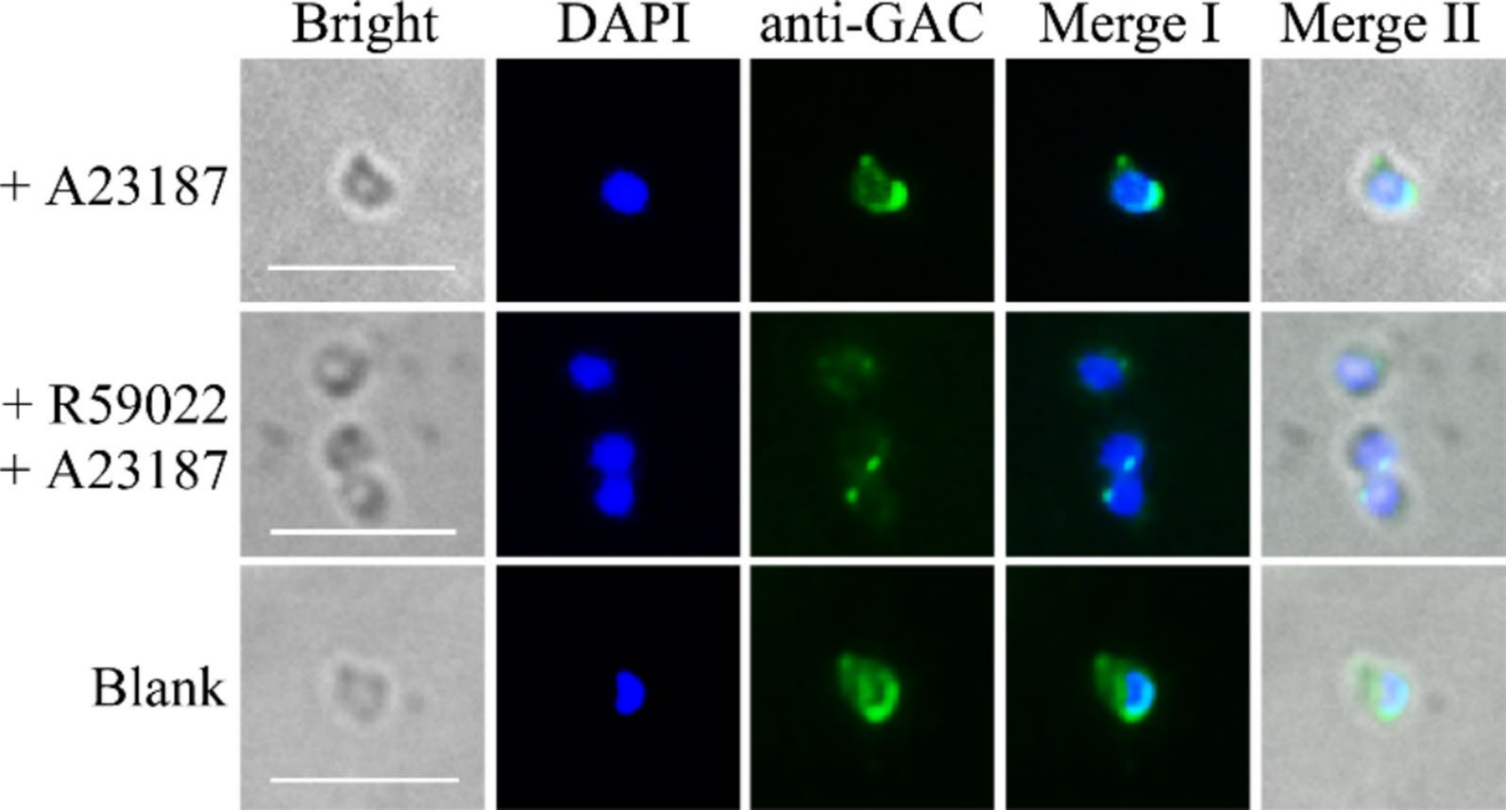
Altered distribution of *Pb*GAC in the presence of the Ca^2+^ ionophore A23187 and the inhibitor of calcium-dependent DGK1 kinase, R59022. Distribution of *Pb*GAC on both the merozoite cytosol and at the apical region is more intense after exposure to A23187. R59022 treatment results in the inhibition of apical to basal transport. Scale bar, 5 μm

The development of parasites depends on calcium-dependent signaling pathways, which influence host-cell invasion and egress from host cells [40]. The Ca^2+^ ionophore A23187 has been previously utilized to study microneme secretion and host-cell invasion in both *T. gondii* and *Plasmodium* parasites [41, 42]. To assess whether *Pb*GAC is associated with calcium levels, merozoites were cultured in the presence or absence of A23187, fixed, and stained with *Pb*GAC antibodies. We observed that *Pb*GAC accumulates at the basal end following parasite egress induced by Ca^2+^ ionophore A23187 (Fig. 3). R59022, an inhibitor of the calcium-dependent DGK1 kinase, is known to inhibit both microneme secretion and parasite motility of *T. gondii* [43]. We examined whether R59022 could affect *Pb*GAC expression and intracellular localization. *P. berghei* parasites were cultured in the presence of A23187 and R59022, fixed, and stained with *Pb*GAC antibodies. In contrast to the effect observed only with A23187, the relocalization of *Pb*GAC from the apical to the basal region was abolished in the presence of the DGK inhibitor R59022 (Fig. 3).

### *Pb*GAC interacts with invasion-associated proteins

**Fig. 4:**
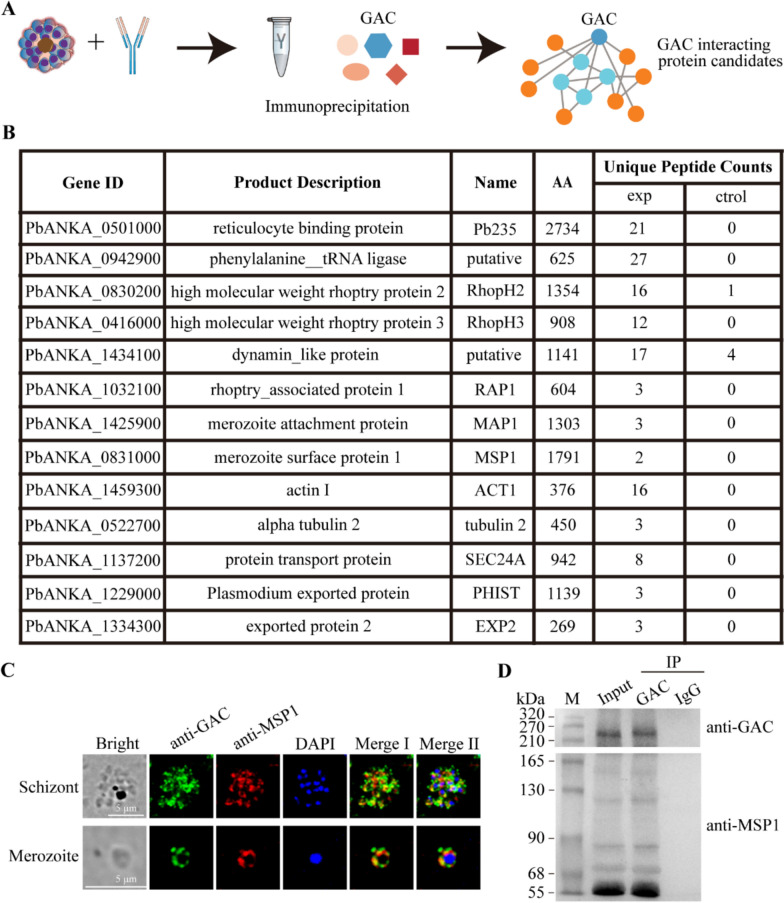
Potential *Pb*GAC interactome. **A**, Schematic overview of the IP. **B**, Proteins identified in the pulldown assay with the *Pb*GAC-specific antibodies. **C**, Colocalization of *Pb*GAC and MSP1 in fixed merozoite and schizont stages. Scale bar 5 μm. **D**, Interactions between *Pb*GAC and MSP1 determined by coimmunoprecipitation (co-IP) and western blotting

To identify potential *Pb*GAC-interacting proteins, we performed a pull-down assay using rat *Pb*GAC-specific antibodies with proteins from synchronized *P. berghei* schizonts, followed by mass spectrometric identification (Fig. 4a). An IgG from a nonimmunized rat was used as a negative control antibody. Over 300 proteins were identified in the sample with the *Pb*GAC-specific antibodies (Supplementary Table S2). Among the proteins identified, reticulocyte binding protein (gene ID no. PbANKA_0501000), high molecular weight rhoptry protein 2 (RhopH2, gene ID no. PbANKA_0830200), RhopH3 (gene ID no. PbANKA_0416000), actin I (gene ID no. PbANKA_1459300), and a transporter protein (SEC24A, gene ID no. PbANKA_1137200) were found to be coprecipitated with *Pb*GAC. Other proteins such as the merozoite attachment protein (MAP1, gen ID no. PbANKA_1425900), the merozoite surface protein 1 (MSP1, gene ID no. PbANKA_0831000), *Plasmodium* exported protein (PHIST, gene ID no. PbANKA_1229000), and the exported protein (EXP2, gene ID no. PbANKA_1334300) also interacted with *Pb*GAC (Fig. 4b). Immunofluorescence with both *Pb*GAC- and MSP1-specific antibodies confirmed the colocalization of the two molecules on the merozoite surface and the potential interaction (Fig. 4c). To further confirm the interaction between MSP1 and *Pb*GAC, we performed co-immunoprecipitation (IP) experiments using *Pb*GAC antibody as bait. The result showed that MSP1 and *Pb*GAC interact (Fig. 4d). These findings provide compelling evidence for the involvement of *Pb*GAC in interactions with *P. berghei* merozoite proteins associated with erythrocyte invasion.

### *Pb*GAC specifically binds heparin

**Fig. 5:**
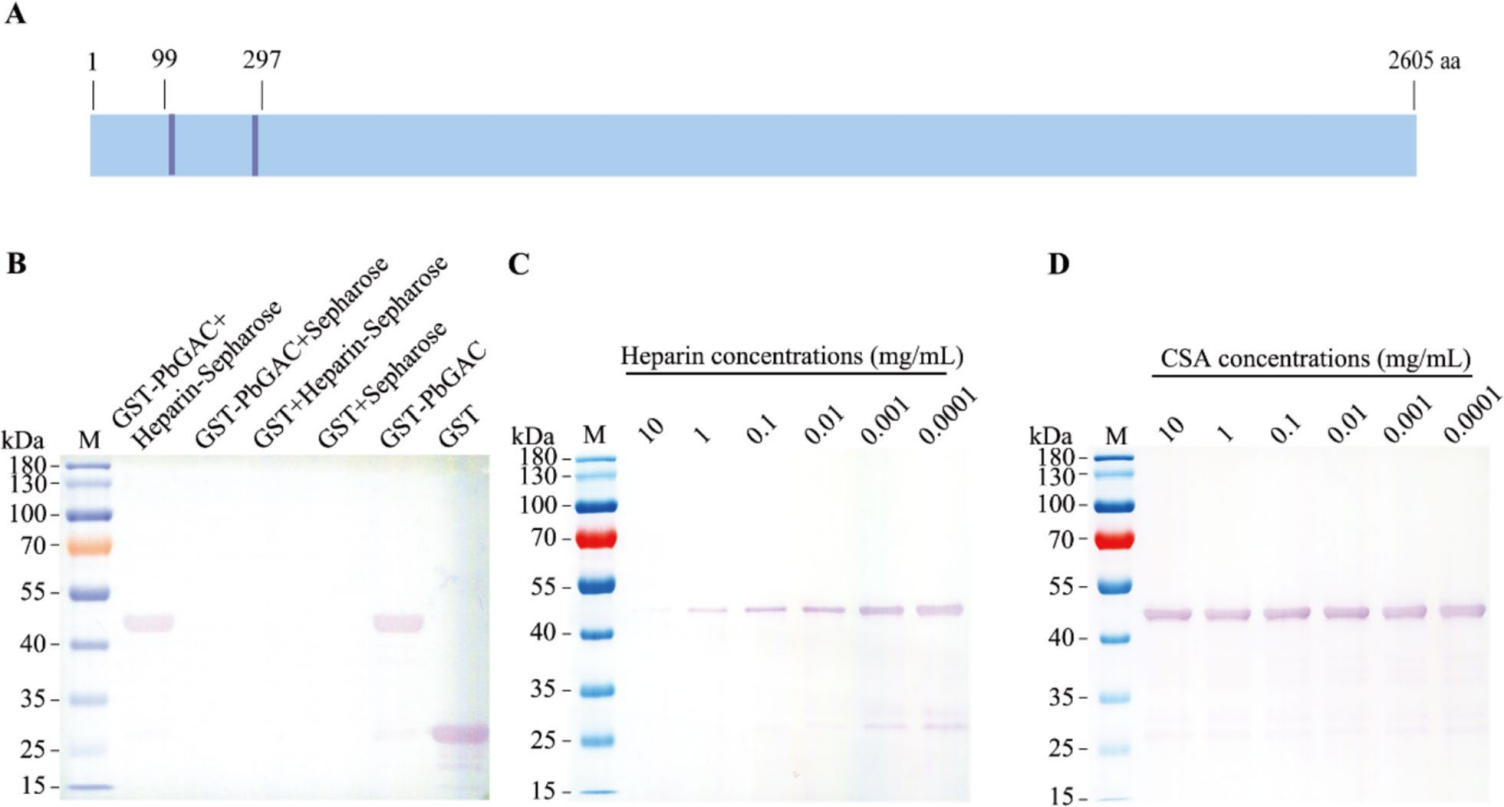
GST-*Pb*GAC specifically binds to heparin. **A**, *Pb*GAC is 2605 amino acids in length, GST-*Pb*GAC contains two heparin-binding motifs (purple). **B**, The heparin-binding capability of GST-*Pb*GAC was assessed via western blotting utilizing an anti-GST antibody. Only GST-*Pb*GAC binds to heparin–sepharose, in contrast to sepharose alone. GST did not bind to heparin–sepharose. **C**, The binding of GST-*Pb*GAC to heparin–sepharose was inhibited by heparin in a concentration-dependent manner. **D**, CSA did not show any inhibitory effect on the binding of GST-*Pb*GAC to heparin–sepharose

We hypothesized that *Pb*GAC may function as an invasion ligand through interaction with heparan sulfate (HS) on the erythrocyte surface, as it possesses several heparin-binding motifs (e.g., XBBXBX and XBBBXXBX; B = basic, X = hydropathic) in the amino acid sequence [44]. To test this hypothesis, we generated recombinant GST-*Pb*GAC and GST fusion proteins for heparin-binding assays. The *Pb*GAC encodes a protein of 2,605 amino acids. To determine *Pb*GAC function in heparin-binding activity, we selected amino acid fragments (amino acids 99–297) that are hydrophilic and contain heparin-binding motif (Fig. 5a). The results indicated that the recombinant protein GST-*Pb*GAC exhibited specific heparin-binding activity, whereas the GST protein did not (Fig. 5b). Furthermore, the *Pb*GAC–heparin interaction could be inhibited by heparin in a dose-dependent manner (Fig. 5c), whereas chondroitin sulfate (CSA) could not (Fig. 5d).

### *Pb*GAC specifically binds to mouse erythrocytes

**Fig. 6:**
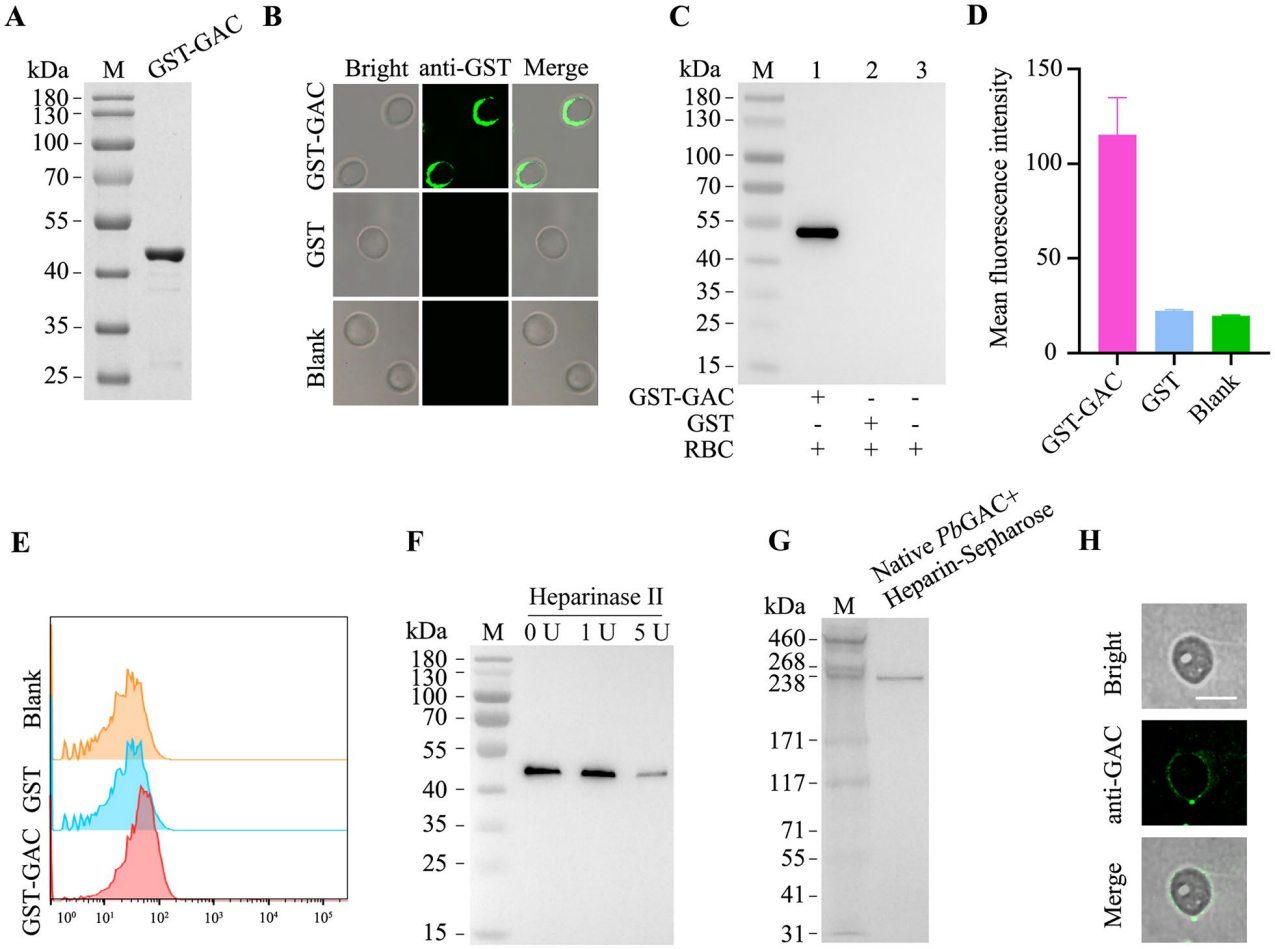
*Pb*GAC binds to mouse erythrocytes. **A**, Purified GST-*Pb*GAC analyzed via SDS–PAGE. **B**, Indirect immunofluorescence assay (IFA) employing an anti-GST antibody as the primary antibody and Alexa Fluor 488 goat anti-mouse IgG (H+L) as the secondary antibody demonstrated GST-*Pb*GAC binding to erythrocytes, with GST serving as the control. Scale bar, 5 μm. **C**, Western blotting with an anti-GST antibody confirmed that only GST-*Pb*GAC binds to erythrocytes. **D**, Flow cytometry analysis revealed a distinct shift in erythrocyte populations bound by GST-*Pb*GAC compared with GST control. **E**, Fluorescence intensity of erythrocytes bound to GST-*Pb*GAC was compared with that of GST and blank controls. **F**, Western blotting showed reduced binding efficiency of GST-*Pb*GAC to erythrocyte surfaces following heparinase II treatment. **G**, Western blotting with an anti-*Pb*GAC antibody confirmed native-*Pb*GAC binding to heparin–sepharose. **H,** Immunofluorescence assay demonstrated native-*Pb*GAC binding to erythrocytes. Scale bar, 5 μm

It has previously been demonstrated that apical organelle-secreted proteins are released into the culture supernatant during host-cell invasion, involving complex interactions between parasite ligands and host receptors [3]. We detected *Pb*GAC in the supernatant (Supplementary Fig. S1). The binding property of *Pb*GAC on erythrocytes was investigated to elucidate its biological functions using GST-*Pb*GAC (Fig. 6a). GST-*Pb*GAC and GST were incubated separately with mouse erythrocytes, and the proteins bound on the erythrocyte surface were analyzed using IFA, western blotting, and flow cytometry. Both IFA and western blotting demonstrated specific binding of GST-*Pb*GAC to the mouse erythrocytes (Fig. 6b, c; Supplementary Fig. S2). This was further confirmed by flow cytometry (Fig. 6d, e), with a gating strategy shown in Supplementary Fig. S3. Conversely, GST alone did not exhibit any binding to the mouse erythrocytes. To confirm that *Pb*GAC mediates heparin interaction, erythrocytes were pretreated with heparinase II before their incubation with GST-*Pb*GAC. Predigestion with heparinase II, which eliminated heparan sulfate from the erythrocyte surface, resulted in a decreased binding affinity of GST-*Pb*GAC to the erythrocytes (Fig. 6f; Supplementary Fig. S4). These findings indicated that GST-*Pb*GAC specifically binds to the erythrocyte surface. To assess whether native *Pb*GAC can bind to heparin, native *Pb*GAC was incubated with heparin–sepharose, and the bound native *Pb*GAC was eluted and analyzed. Native *Pb*GAC was capable of directly binding to heparin (Fig. 6g). Similar to its binding profile with heparin, native *Pb*GAC also bound to mouse erythrocytes (Fig. 6h).

### Mice immunized with recombinant *Pb*GAC exhibited resistance to *P. berghei* infection

**Fig. 7:**
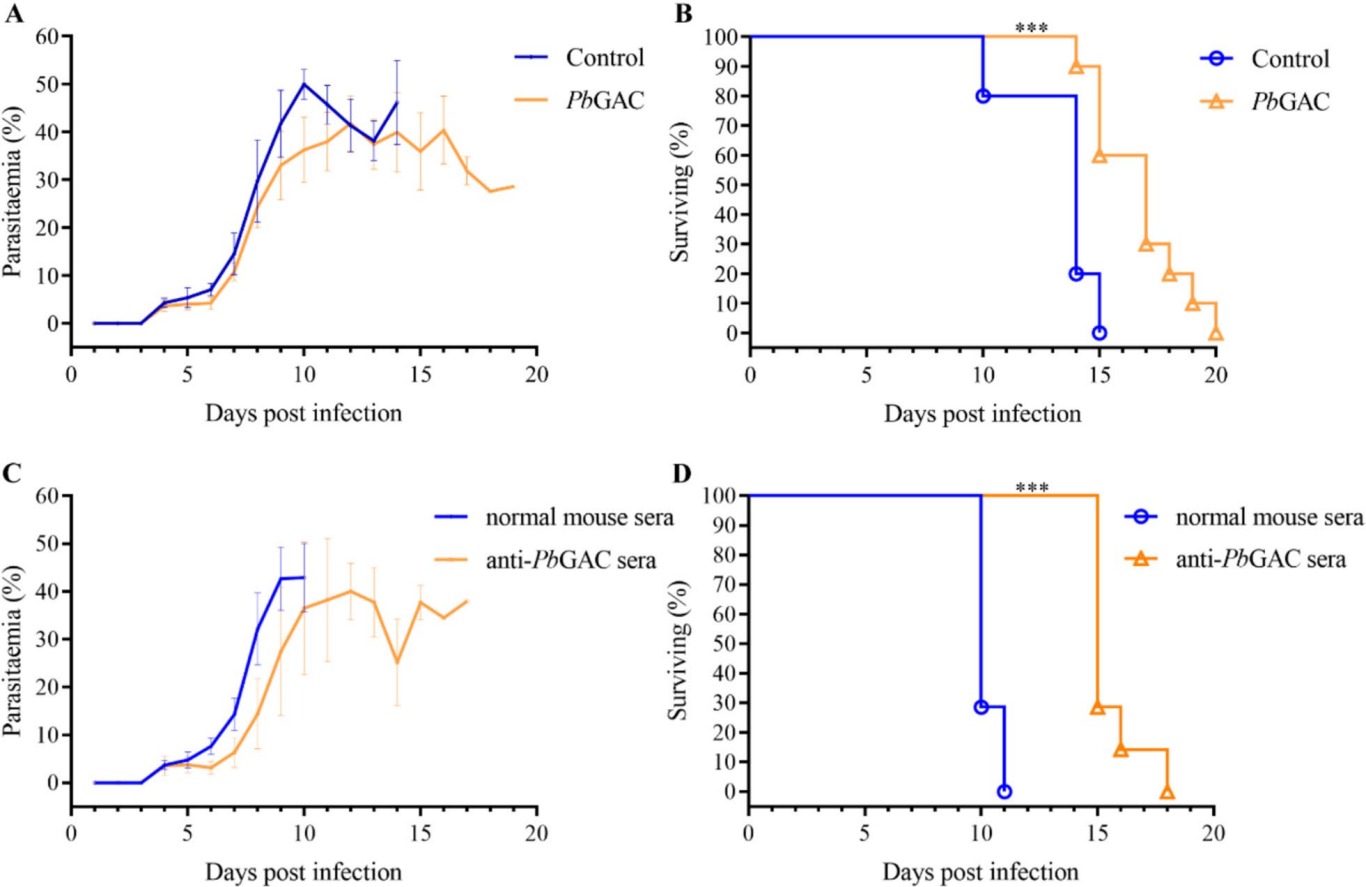
*Pb*GAC-specific antibodies confer protection against *P. berghei* infection. **A**, **B**, Control BALB/c mice not immunized with *Pb*GAC displayed higher parasitemia than *Pb*GAC-immunized mice; the error bars represent standard deviation (SD). *Pb*GAC-immunized mice survived an average of 5 days longer than the control group. **C**, **D**, Mice injected with serum from a normal mouse exhibited higher parasitemia than those receiving anti-*Pb*GAC immune serum. The anti-*Pb*GAC immune serum group survived 5 days longer; the error bars represent SD ^***^P < 0.001. Data were analyzed by log-rank (Mantel–Cox) test

To evaluate whether *Pb*GAC-specific antibodies confer in vivo protection, groups of BALB/c mice (*n* = 10/group) were immunized four times with *Pb*GAC-His in Freund’s adjuvant, with a control group receiving only Freund’s adjuvant. Once serum titers of *Pb*GAC-specific antibodies reached 1:32,000, mice were intraperitoneally injected with 1 × 10^6^ *P. berghei*-infected erythrocytes in 200 μL of 0.9% NaCl. As expected, *Pb*GAC-immunized mice showed prolonged survival (Fig. 7a, b). In addition, two groups received a single intravenous dose of 500 μL immune serum or PBS as a control. The following day, all mice were challenged with 1 × 10^6^ *P. berghei*-infected erythrocytes. Consistent with previous results, mice injected with anti-*Pb*GAC serum exhibited significantly longer survival than the control group (Fig. 7c, d). These findings suggest that *Pb*GAC antibodies confer protection against *P. berghei* infection.

## Discussion

The invasion of host erythrocytes constitutes a hallmark biological process for obligate intracellular parasites such as *Plasmodium* species. Following invasion, merozoites execute a precisely coordinated, multistage invasion process involving preinvasion recognition and active penetration. This intricate mechanism relies on the spatiotemporal regulation of surface-exposed proteins and invasion-associated organellar components, including rhoptries and micronemes [3]. Many of these proteins are anchored via glycosylphosphatidylinositol (GPI) linkages [45]. Several merozoite invasion ligands have been characterized, notably the merozoite surface proteins (MSPs), which constitute a significant component of the merozoite surface coat [3, 9]. Among these, GPI-anchored merozoite surface protein 1 (MSP1) is the largest, forming a dimer exceeding 500 kDa, and is the most abundant [9, 17, 46, 47]. Although MSP1’s precise role remains to be elucidated, the binding affinity of its fragments MSP1-42 and MSP1-33 to heparin imply that heparin-like glycosaminoglycans serve as receptors facilitating erythrocyte invasion [24]. Furthermore, among the key molecular players, MAP1 has been characterized as heparan sulfate (HS)-binding ligand that mediates the initial adhesion of the merozoites to the erythrocyte surface [18], while the PfRh/EBL protein families orchestrate invasion signaling through interactions with erythrocyte receptors such as glycophorins A/B/C and complement receptor 1 [11, 19]. The irreversible host–parasite interface is stabilized by the AMA1–RON complex interaction, where transmembrane RON2 anchors in the erythrocyte membrane to engage merozoite-surface AMA1 [23]. We have revealed that heparin-binding proteins of both *P. falciparum *and *P. berghei* are critically involved in erythrocyte invasion [18, 48], highlighting protein–glycosaminoglycan interactions as potential therapeutic targets. This study suggests that *Pb*GAC plays a role in the binding of HS during erythrocyte invasion.

*Pb*GAC is predominantly localized at the apical region and in the cytosol of *P. berghei* merozoites, suggesting its potential involvement in host–pathogen interactions. *Plasmodium falciparum* reticulocyte-binding protein homolog 1 (PfRH1) and erythrocyte-binding protein EBA175 are critical mediators of erythrocyte invasion by the parasite [19, 49, 50]. Unlike other EBA/PfRh ligands that exhibit functional redundancy [51], PfRH1 specifically triggers calcium signaling pathways, leading to the release of EBA175 and facilitating junction formation between the erythrocyte and the parasite [52]. We analyzed calcium flux triggered by A23187. Calcium flux-triggered microneme secretion represents a critical regulatory node for apicomplexan motility and invasion [53]. Pharmacological modulation using Ca^2+^ ionophore A23187 induced *Pb*GAC translocation to the basal pole during egress, a process disrupted by DGK1 inhibition via the R59022 compound [43]. This parallels *Tg*GAC dynamics, suggesting conserved regulatory mechanisms. Calcium ions are required at two critical stages during the intraerythrocytic asexual life cycle of *Plasmodium*: during the initial invasion of the red blood cell and the initiation of egress [20, 54, 55]. In most cases, the Ca^2+^ flux was an excellent indicator of successful invasion. The transient, motility-dependent basal accumulation of *Pb*GAC and observed immune protection in *Pb*GAC-His vaccinated mice (Fig. 7) further underscore its functional significance. Our immunoprecipitation studies reveal *Pb*GAC’s interactome, encompassing MSP1, MAP1, RhopH complex components, RAP1, and actin-regulatory proteins. This molecular synergy aligns with emerging models of moving junction formation [56, 57], where apical-to-basal protein translocation facilitates mechanical penetration.

Several studies have demonstrated that heparin-like molecules inhibit erythrocyte invasion by blood-stage *Plasmodium* spp. [24, 58, 59]. These findings imply that the heparin-like molecules play an important role in invasion inhibition and possess potential as novel antimalarial therapeutics. Nonetheless, only a limited number of merozoite proteins have been characterized for their capacity to bind heparin-like molecules and facilitate erythrocyte invasion. Surface antigens, including merozoite surface protein 1 (MSP1), are hypothesized to mediate initial erythrocyte attachment [3], potentially serving as receptors for heparin-like molecules. Kobayashi et al. reported that heparin binds at the apical pole of the merozoite surface, with multiple heparin-binding proteins localized preferentially within apical organelles [28]. Our data indicate that *Pb*GAC is expressed during the merozoite stage, with partial localization to the merozoite apical region and cytosol. Furthermore, we demonstrated direct binding between *Pb*GAC and heparin-agarose beads, suggesting that *Pb*GAC binds to the erythrocyte surface. Immunofluorescence assays, western blotting, and flow cytometry confirmed that *Pb*GAC directly binds to the erythrocyte surface. In addition, the binding of GST–GAC with erythrocytes was sensitive to heparinase II pretreatment, indicating dependence on heparan sulfate. The initial attachment is a pivotal step requiring both interaction specificity and cellular process efficiency. Our findings suggest that *Pb*GAC fulfills the complex molecular criteria for initial erythrocyte attachment: it is localized on the merozoite cytosol and apical region, highly conserved across the *Plasmodium* spp., and remarkably specific for HS binding.

## Conclusions

Our findings demonstrate a novel protein, *Pb*GAC, predominantly expressed in the *P. berghei* merozoite cytosol and the apical region, which is associated with erythrocyte invasion. The distribution of *Pb*GAC in the parasite is modulated by calcium ions. Furthermore, *Pb*GAC specifically interacts with the heparan sulfate receptor on erythrocytes, implying a common mechanism of malarial parasite invasion. The significance of *Pb*GAC in the invasion process is evidenced by its strong interaction with several invasion-associated proteins and the protective effect of specific antibodies against parasite invasion. These mechanistic insights not only advance our understanding of *Plasmodium* invasion biology but also establish an experimental framework for the systematic functional characterization of *P. falciparum* virulence factors, potentially accelerating the development of antimalarial therapeutics targeting host–pathogen interactions.

## Supplementary Information

Additional file 1: Fig. S1. *Pb*GAC is secreted into the supernatant. The culture supernatant (SN) was stained with anti-*Pb*GAC antibody and analyzed by western blotting.

Additional file 2: Fig. S2. The binding of GST-*Pb*GAC to erythrocytes in SDS-PAGE gel.

Additional file 3: Fig. S3. Flow cytometry gating strategy.

Additional file 4: Fig. S4. The effect of heparinase II treatment on the binding of GST-*Pb*GAC to erythrocytes in SDS-PAGE gel.

Additional file 5: Table S1: Primers used for *Pb*GAC cloning.

Additional file 6: Table S2: Identification of proteins interacting with *Pb*GAC.

## Data Availability

The proteomic analysis data have been deposited in the ProteomeXchange Consortium (https://proteomecentral.proteomexchange.org) via the iProX partner repository with the dataset identifier PXD061524.

## Abbreviations

*Pb*ANKA: *Plasmodium berghei* ANKA
HS: Heparan sulfate
CSA: Chondroitin sulphate A
SDS–PAGE: Sodium dodecyl sulphate–polyacrylamide gel electrophoresis
RPMI: Roswell Park Memorial Institute
DAPI: 2′,6-diamidino-2-phenylindole
MSP: Merozoite surface protein
RBC: Red blood cell
EBL: Erythrocyte binding-like
RBL: Reticulocyte binding-like
AMA: Apical membrane antigen
GAC: Glideosome-associated connector
TRAP: Thrombospondin anonymous repeat protein
EBA: Erythrocyte binding-like protein
PfRh: Reticulocyte-binding protein
MAP: Merozoite attachment protein
PBS: Phosphate buffer saline
