# Supplementary figures and images for: The Plasmodium berghei merozoite protein PbGAC is critically involved in erythrocyte binding during invasion
Source: Parasit Vectors. 2025 Oct 28;18:432. doi: 10.1186/s13071-025-07067-5 (PMC12570789; doi:10.1186/s13071-025-07067-5)

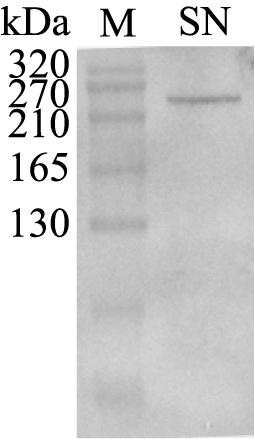

Supplement: Supplementary file 1 — Additional file 1: Fig. S1. PbGAC is secreted into the supernatant. The culture supernatant (SN) was stained with anti-PbGAC antibody and analyzed by western blotting. [file 13071_2025_7067_MOESM1_ESM.tif]

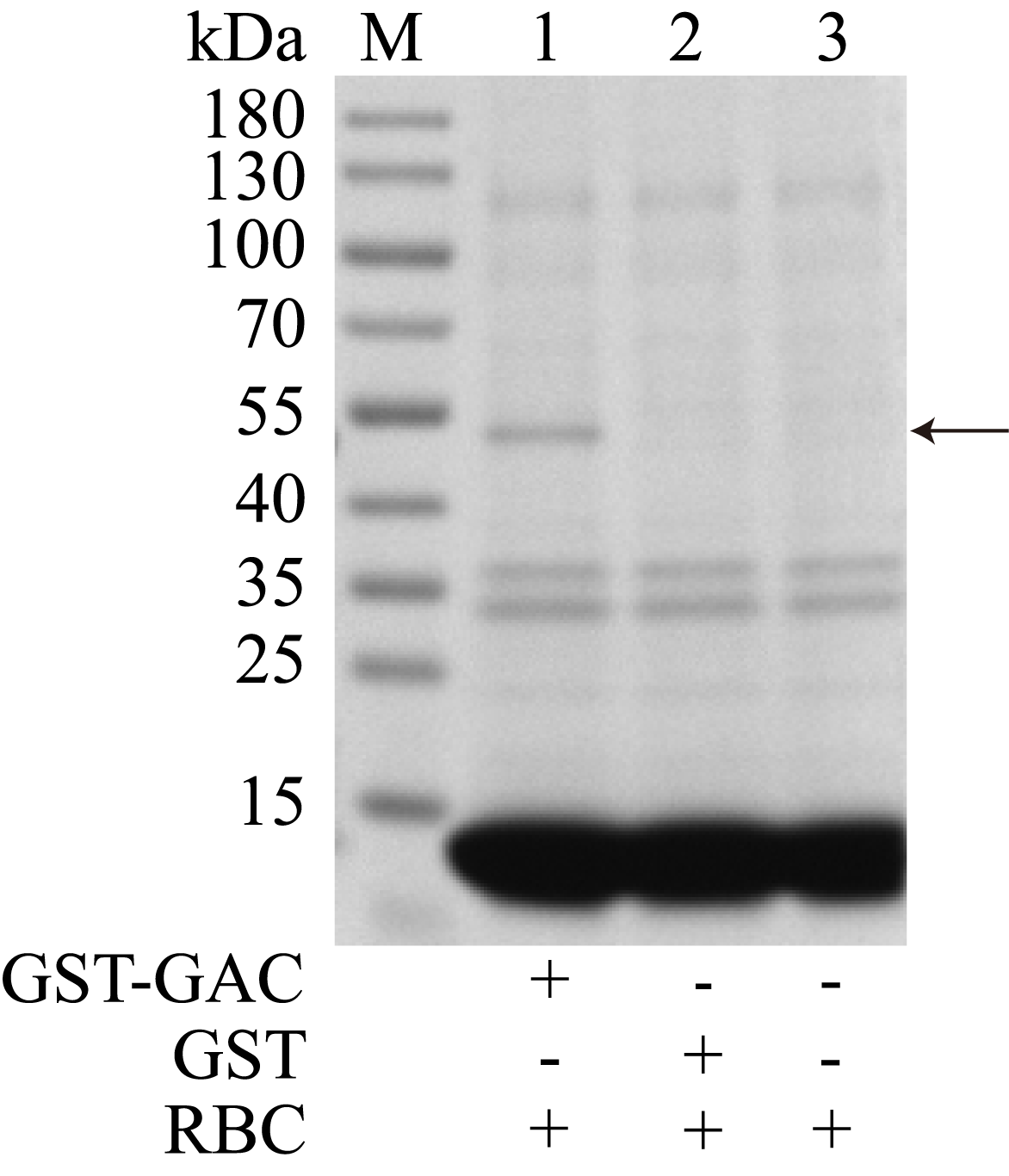

Supplement: Supplementary file 2 — Additional file 2: Fig. S2. The binding of GST-PbGAC to erythrocytes in SDS-PAGE gel. [file 13071_2025_7067_MOESM2_ESM.tif]

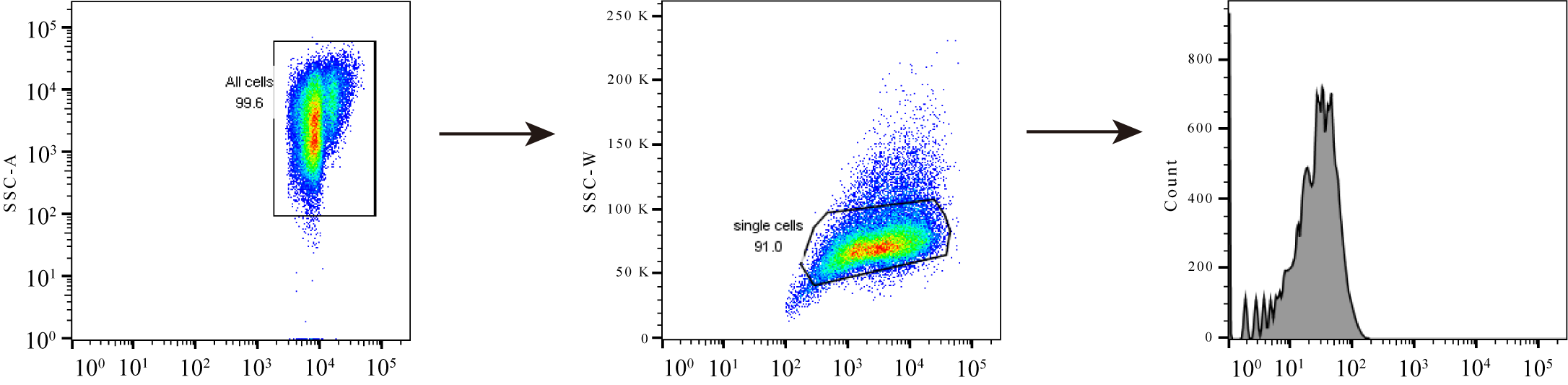

Supplement: Supplementary file 3 — Additional file 3: Fig. S3. Flow cytometry gating strategy. [file 13071_2025_7067_MOESM3_ESM.tif]

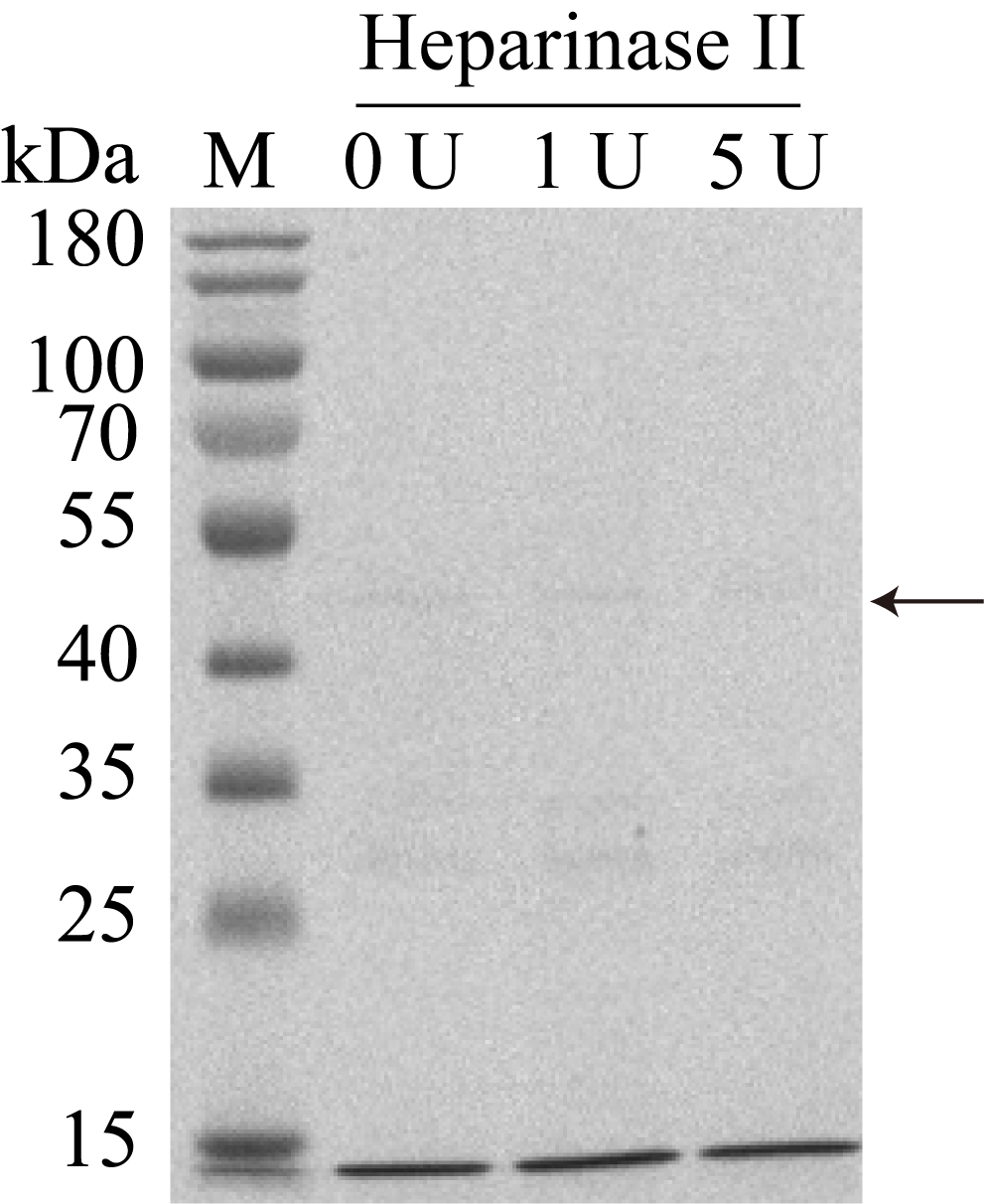

Supplement: Supplementary file 4 — Additional file 4: Fig. S4. The effect of heparinase II treatment on the binding of GST-PbGAC to erythrocytes in SDS-PAGE gel. [file 13071_2025_7067_MOESM4_ESM.tif]
